# Supplementary material for: Endocrine Disruptors and Endometrial Cancer: Molecular Mechanisms of Action and Clinical Implications, a Systematic Review
Source: Int J Mol Sci. 2022 Mar 9;23(6):2956. doi: 10.3390/ijms23062956 (PMC8953483; doi:10.3390/ijms23062956)
Supplement: Supplementary file 1 [file ijms-23-02956-s001.zip › ijms-1610298-supplementary.pdf]

Methods

The present systematic review was performed according to the Systematic Reviews and Meta-Analyses (PRISMA) guidelines. All in vivo, in vitro studies analyzing the interaction of EDCs with EC, were searched. A search was performed on the PubMed®/MEDLINE® database and included the last 20 years of publication (2000–2020). Only studies in English were included. The search was carried out in September 2021. The terms used for the search were: "Endocrine Disrupters" OR "Heavy Metals", OR "Bisphenol A" Perchlorate ", OR "Perfluoroalkyl" OR "Polybrominated diphenyl ethers" OR "Polychlorinated biphenyls " OR "Phthalate" AND "Endometrial cancer".

A total of 473 results were obtained. Twenty-three duplicates were excluded. A total of 418 articles were excluded after reading the abstract and title. Thirty-four articles remained for reading the full texts. Among these studies, eight were excluded. Six of the studies were excluded because studies did not perform EDCs analysis, while three studies did not have EC data extrapolation. A total of 25 articles were finally included in the systematic review. The study-selection process is summarized in the PRISMA flow chart (Figure S1).

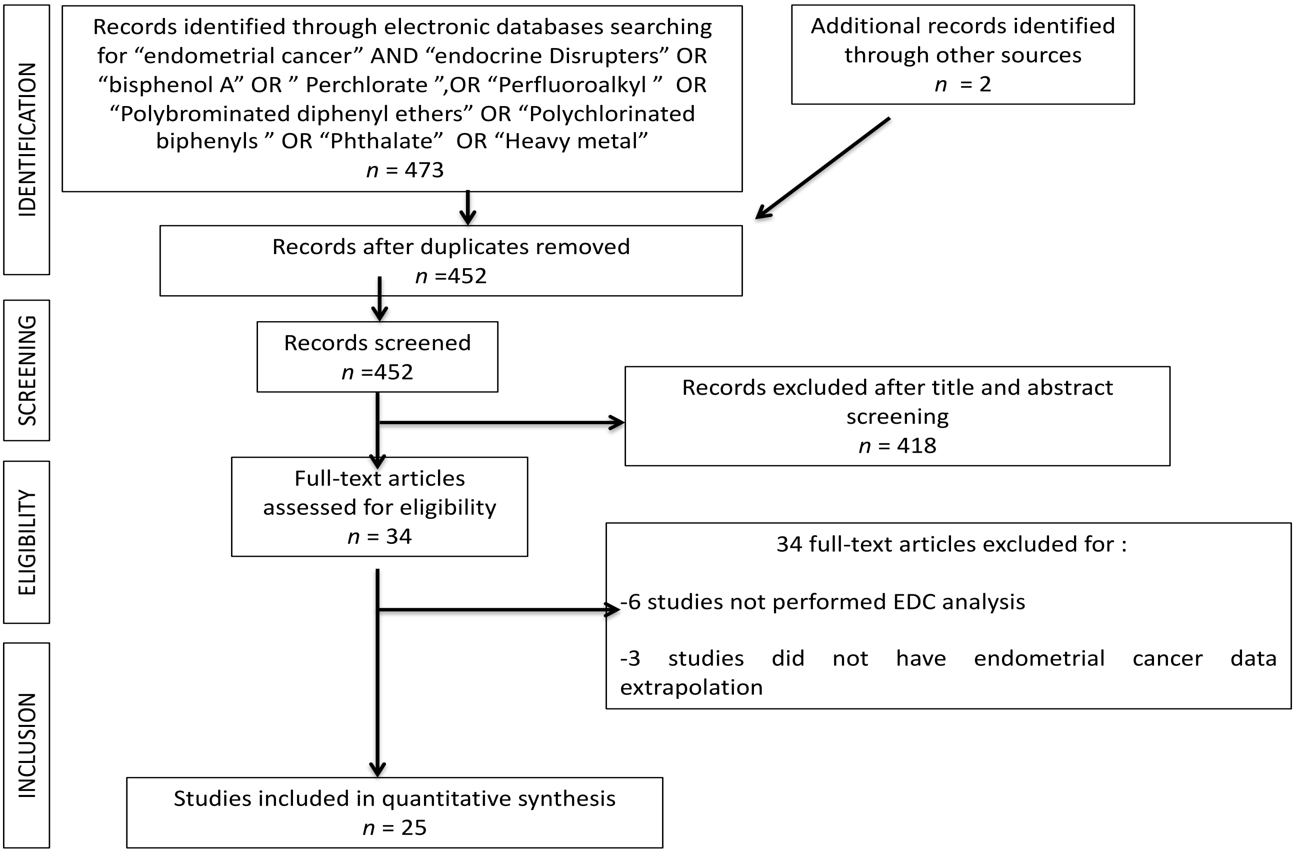

Figure S1. PRISMA flow-chart.
